# LC–MS/MS method for the quantification of masitinib in RLMs matrix and rat urine: application to metabolic stability and excretion rate

**DOI:** 10.1186/s13065-017-0365-2

**Published:** 2017-12-22

**Authors:** Sawsan M. Amer, Adnan A. Kadi, Hany W. Darwish, Mohamed W. Attwa

**Affiliations:** 1 Analytical Chemistry Department, Faculty of Pharmacy, Cairo University, Kasr El-Aini St., Cairo, 11562 Egypt; 2 Department of Pharmaceutical Chemistry, College of Pharmacy, King Saud University, P.O. Box 2457, Riyadh, 11451 Kingdom of Saudi Arabia

**Keywords:** Masitinib, Tandem mass spectrometry, Quantification, Metabolic stability, Rat liver microsomes, Rate of urine excretion

## Abstract

Masitinib (MST) is a selective tyrosine kinase inhibitor. Validated liquid chromatography tandem mass spectrometric method (LC–MS/MS) was developed for the quantification of MST in rat liver microsomes (RLMs) matrix. The developed method was applied to metabolic stability and excretion rate studies. Reversed phase liquid chromatography was used for resolution of MST and bosutinib (IS) using C_18_ (50 mm × 2.1 mm, 1.8 μm). Binary solvent system consisted of 35% solvent A (0.1% formic acid in H_2_O, pH: 3.2) and 65% solvent B (acetonitrile) used as mobile phase at flow rate of 0.25 mL with a total run time of 5 min. Injection volume was 5 µL. Generation of ions was done in positive ESI source and quantification of MST and IS were done using MRM mode. The developed method showed a linearity in the range of 5–200 ng/mL (r^2^ ≥ 0.9992) with *LOQ* and *LOD* of 0.25 and 0.76 ng/mL in RLMs. The intra- and inter-day precision and accuracy ranged from 0.95 to 1.49 and − 5.22 to 1.13%, respectively in RLMs. Rate of disappearance of MST during incubation with RLMs was almost linear allover incubation time. In vitro t_1/2_ was 50.38 min and CL_in_ was 3.11 ± 0.2. The developed method was applied also to measure the rate of masitinib excretion in rat urine. The method can used for further pharmacokinetic studies of MST.

## Introduction

**Fig. 1:**
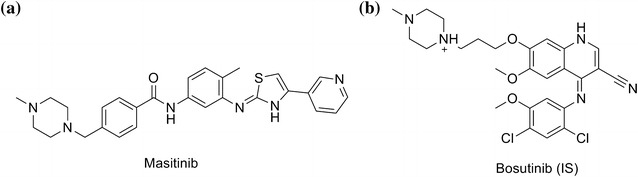
Chemical structure of **a** masitinib (MST) and **b** Bosutinib (IS)

MST (Fig. 1) is a selective TKIs. MST is registered for the treatment of mast cell tumors in dogs under the trade name of Masivet in Europe (since 2009) and Kinavet in USA (since 2011) [1, 2] _ENREF_11_ENREF_2. It acts selectively targeting mainly wild type forms and mutated c-Kit R, PDGFRα/β, Lck, LYn, FGFR3 and FAK. It is considered the first approved anticancer veterinary therapy for the treatment of unresectable canine mast cell tumors (CMCTs), which harbors activating c-KitR mutations at dose of 12.5 mg/kg per day [3] _ENREF_1. It is more active and selective against KIT than imatinib in in vitro studies [4, 5].

Metabolic stability of MST was evaluated based on rate of disappearance during incubation with RLMs matrix [6]. In vitro half-life (t_1/2_) and intrinsic clearance (CL_int_) were used to express MST metabolic stability. Based on MST metabolic stability data, secondary pharmacokinetic parameters such as hepatic clearance (CLH), bioavailability and in vivo t_1/2_ can be calculated which is important for creating in vivo–in vitro correlation for proper metabolic stability study. If the studied drug is rapidly metabolized, in vivo bioavailability will be low [7].

Upon literature review, there was no publication about masitinib analysis. An LC–MS/MS technique was developed in the current study for quantification of MST concentration in RLMs matrix. The current method was fast, sensitive, specific and reproducible. Moreover, it is considered the first validated LC–MS/MS for assaying MST. The developed method is applied for assessing the metabolic stability of MST in RLMs matrix. The developed method was applied also to measure the rate of masitinib excretion in rat urine.

## Experimental

### Chemicals and reagents

All chemicals unless otherwise stated were mentioned in a previous published article [8]. Masitinib and bosutinib were procured from LC Laboratories (Woburn, MA, USA). Preparation of RLMs was done in-house using Sprague Dowely rats [9].

### Chromatographic conditions

Chromatographic separation for the MST (analyte) and bosutinib (IS) was done using Agilent LC–MS/MS (6410 QqQ) which has been described in detail elsewhere [8]. Reversed phase liquid chromatography was used for separation of MST and bosutinib (IS) using C18 (50 mm × 2.1 mm, 1.8 μm). Binary solvent system consisted of 35% solvent A (0.1% formic acid in H2O, pH: 3.2) and 65% solvent B (acetonitrile) used as mobile phase at flow rate of 0.25 mL with a total run time of 5 min. Injection volume was 5 µL. Temperature of the column was fixed at 22 °C. Mass spectrometric parameters and chromatographic conditions for MST and bosutinib were optimized to accomplish the best resolution in a very short time and the highest response. Nitrogen generator was adjusted to generate low purity nitrogen for electrospray ionization (ESI) source (drying gas) at a flow rate of 12 L/min and high purity nitrogen for collision cell (collision gas) at a pressure of 50 psi. ESI source temperature and capillary voltage were adjusted at 350 °C and 4000 V respectively. Agilent triple quadrupole was controlled by Mass Hunter software. Multiple reaction monitoring (MRM) of the transition 499 → 399 for MST and 530 → 113 and 530 → 141 for bosutinib (IS) data was used for quantification. Fragmentor voltage was set to 135 V with collision energy 20 for MST and 135 and 140 V with collision energy of 15, 20 for bosutinib.

### Preparation of working standard solutions

Concentration of 1.0 mg/mL of MST was dissolved in DMSO then 1 mL of this solution was diluted ten fold using mobile phase to prepare working standard 1 (WS1, 100 µg/mL). One milliliter WS1 was diluted tenfold using mobile phase to prepare working standard 2 (WS2, 10 µg/mL). Concentration of 0.1 mg/mL of IS was prepared in DMSO then 200 µL of this solution was diluted 50-fold using mobile phase to prepare working IS (WI, 2 µg/mL).

### Calibration curve and sample preparation

Proper volumes of MST WS2 (10 µg/mL) were diluted with a suitable volume of RLMs matrix to prepare eight concentrations: 5, 10, 15, 20, 50, 100, 150 and 200 ng/mL in a final volume of 1 mL. Fifteen, fifty and one hundred fifty were chosen as low quality control (LQC), medium quality control (MQC) and high quality control (HQC), respectively. Two milliliter of ACN were added for protein precipitation. Centrifugation (14,000 rpm, 12 min and 4 °C) was done to remove precipitated proteins. Supernatants were detached and filtered using 0.22 µm syringe filters. Fifty microliter of WI solution was added to 1 mL of calibration standards. Blank samples were prepared in the same procedure without adding MST. Blank samples were tested to confirm the absence of any interference with MST and IS at their retention times. Calibration curve was created for spiked RLMs samples by plotting the peak area ratio of MST to IS (*y* axis) against MST nominal concentrations (*x* axis).

### Validation of the method

Validation of the current method was done following the guidelines of FDA [10] and the general recommendations of ICH [11, 12].

#### Specificity

Six separate blank RLMs matrix samples were treated with the previously mentioned extraction technique. Chromatograms were screened for any interferences peaks at retention times of MST or IS, MRM mode in the mass detector was used to raise the specificity of the current method. Post time (2 min.) and injector washing were used to minimize carryover effects.

#### Linearity and sensitivity

Evaluation of the linearity and sensitivity of the current method was done using six calibration curves. Statistical least square method was used to analyze the data. ICH guidelines were used for calculation of *LOD* and *LOQ* [11], based on the intercept standard deviation and slope of the calibration plot through the following equation.

$$ LOQ\;OR\;LOD = mS{\big/}n$$

where *m* equals 3.3 for *LOD* and 10 for *LOQ*, *S* is the standard deviation of the intercept, and *n* is the slope.

#### Precision and accuracy

The basis of calculation of intra-day and inter-day precisions and accuracies was the analysis of RLMs matrix samples’ spiked with MST at QC levels in 1 day and three consecutive days, respectively. Percentages error and percentages relative standard deviation were utilized for expressing accuracy and precision. The equation of calculations were mentioned Percentages RSD = (SD/Mean) ×100 and percentages error = [(average measured concentration − expected concentration) / expected concentration] ×100].

#### Stability

For assessing MST stability in RLMs matrix, six replicates of MST QC samples were analyzed under different storage circumstances. Freshly prepared RLMs calibration curves were used for computing of accuracy and precision values were carried out using. Eight hours of MST QC samples storage at room temperature was used to evaluate MST bench-top stability. Three freeze–thaw cycles were used to estimate MST stability of spiked QC samples after freezing them at – 80 °C and thawing them at ambient temperature. Evaluation of MST stability was done by analyzing the spiked QC samples that were kept at 4 °C for 1 day and stored − 20 °C for 1 month.

### Metabolic stability of MST

After incubation with RLMs matrix, tracking the decrease in MST concentration was performed utilizing the current method. Incubations were done for 1 µM MST with 1 mg/mL microsomal proteins, and 1 mM NADPH in phosphate buffer (pH 7.4) containing 3.3 mM MgCl_2_ in 1 mL. NADPH was used to initiate the metabolic reaction and 2 mL of ACN was used to terminate it. Metabolic reaction was terminated at specific time intervals (0, 2.5, 5, 10, 15, 20, 40 and 50 min). Centrifugation (14,000 rpm for 12 min at 4 °C) was done to remove precipitated proteins. Supernatants were filtered using 0.22 µm syringe filter. The filtered samples were diluted twofold with mobile phase to make the final conc. of MST in the linear dynamic range of the current method. One milliliter of the diluted solution was transferred to HPLC vial then IS WI (50 µL) was added. Injection volume was 5 µL into the LC–MS/MS system. Concentrations of MST in RLMs matrix were computed from the regression equation of freshly constructed calibration curve using peak area ratios of MST and IS.

### Rate of MST excretion in rat urine

The current method was also applied to measure MST in rat urine at specific time intervals after single oral dose of 33.3 mg/Kg using oral gavage. Six Male Sprague–Dawley rats were brought from college of pharmacy animal house, King Saud University (Riyadh, KSA). Rats were housed individually in special purpose cages. Masitinib was dissolved in (4% DMSO, 30% PEG 300, 5% Tween 80, HPLC H_2_O) for oral dosing of rats. Dose of masitinib in rats were calculated using a specific conversion equation of drugs between animals [13–15]. Kinavet-CA1 dose in dogs was 10 mg/kg. So the dose for rat were 33.3 mg/Kg. Blank urine was collected before MST administration. Urine samples were collected at 6, 12, 18, 24, 48, 72 and 96 h. following masitinib dosing and then filtered over 0.45 µm syringe filters for removal of particulate matters in the urine. Two milliliter were taken from each sample, equal amount of ACN was added and then strongly shaken for 1 min and the mixture was stored at 4 °C overnight. Two solvent layers were formed, an upper ACN layer and a lower aqueous layer. Upper ACN layer was diluted twenty timed with mobile phase to make the final conc. of MST lied in the linearity range. IS (50 µL) was added to 1 mL of the diluted solution. Five microliter of this solution was injected into the LC–MS/MS system. Control urine samples obtained from rats prior to drug dosing were prepared in a similar way to the above mentioned method.

## Results and discussion

### Optimization of chromatographic and mass spectrometric parameters

**Fig. 2:**
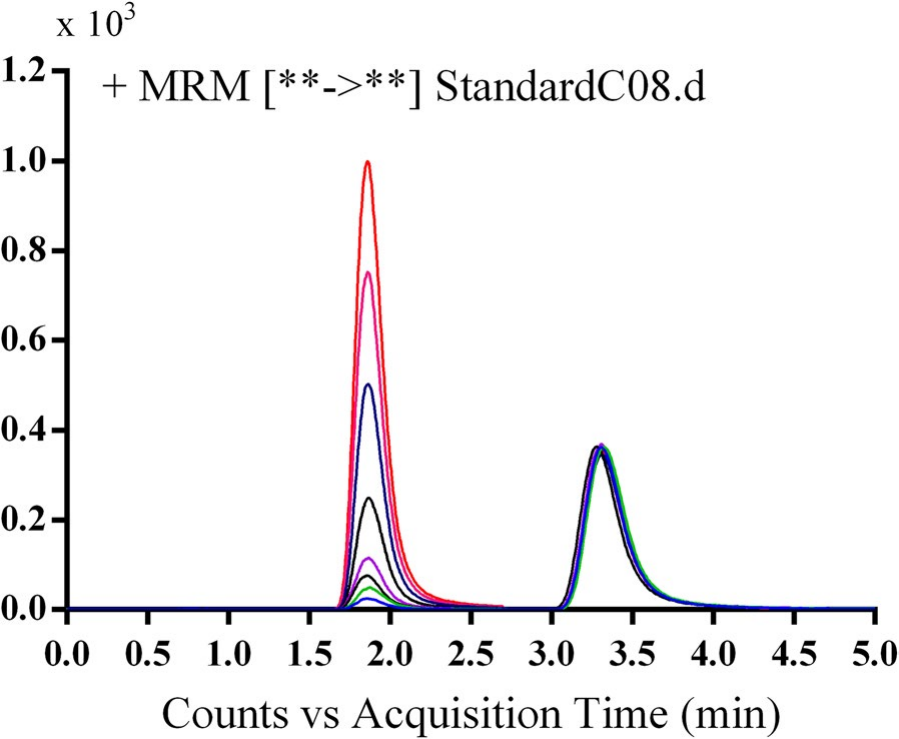
TIC chromatogram of MRM of MST (5–200 ng/mL) and IS (100 ng/mL)

Several experiments were performed to attain the best mass response by optimizing all chromatographic and mass spectrometric parameters so as to enhance the resolution and sensitivity. Mobile phase system consisted of ACN and 0.1% formic acid in HPLC H_2_O (pH ~ 3.2) with a ratio of (35:65, v/v) flowing at 0.25 mL/min. MST and IS were detected at retention times of 1.9 and 3.3 min, respectively. The run time of the current method was 5 min. MST and IS peaks were well resolved, with no carryover in any blank matrix (RLMs) sample or MST-free standard (blank + internal standard). A representative chromatogram of standard solutions of a calibration curve is shown in Fig. 2.

**Fig. 3:**
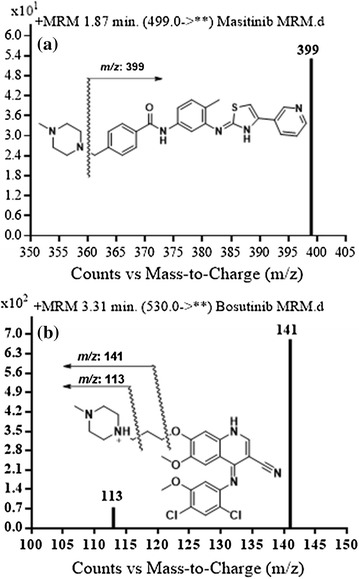
MRM mass spectra of **a** MST and **b** Bosutinib (IS)

The positive MS scan showed molecular ion peaks at m/z 499 and at m/z 530 for MST and IS, respectively. Fragmentation of MST at *m/z* 499 gave one daughter ion of at m/z 399. Similarly, fragmentation of IS ion at *m/z* 530 gave daughter ions at *m/z* 113 and at *m/z* 141. Those ions were chosen for the MRM mode of MST and IS in the current method (Fig. 3).

### Method validation

#### Specificity

The current method was found to be specific because no interference from RLMs matrix constituents at the retention time of MST and/or IS. No carry over effect was observed in the mass detector. MST and IS peaks were well separated with retention times of 1.9 and 3.3 min., respectively.

#### Linearity and sensitivity

The current method was rugged and sensitive for MST analysis in RLMs matrix. The linear range was 5–200 ng/mL, with r^2^ ≥ 0.9992 in RLMs matrix. The regression equation of the calibration curve of MST in RLMs was y = 1.0439x − 0.6999. The *LOD* and *LOQ* were 0.25 and 0.76 ng/mL in RLMS. The good linearity of the current method was indicated by the high r^2^ value. The validity of the calibration points to construct a calibration curve was indicated by low values of SD of the intercept and the slope.

Concentration point RSD values (six replicates) was found to be less than 2%. Back calculation of calibration and quality control standards of MST in RLMs matrix was done to ensure the best performance of the current method. The precision and accuracy for MST in RLMs samples were ranged from 0.28 to 2 and − 3.06 to 2.89%, respectively (Table 1). The mean % recoveries of MST were 99.58 ± 2.3% in RLMs.

**Table 1:** Data of back-calculated MST concentration of the calibration standards

Nominal concentration (ng/mL)	Mean^a^	Standard deviation (SD)	Precision (RSD%)	Accuracy (RE%)
5	5.14	0.07	1.38	2.89
10	9.83	0.11	1.16	− 1.72
15	14.61	0.06	0.44	− 2.59
20	19.72	0.39	2.00	− 1.41
50	48.47	0.28	0.58	− 3.06
100	102.04	0.46	0.45	2.04
150	152.73	0.64	0.42	1.82
200	197.38	0.54	0.28	− 1.31

^a^Average of six determinations

#### Precision and accuracy

Intra- and inter-day precision and accuracy of MST QC samples were used to confirm the reproducibility of the current method. Accuracy and precision was expressed in terms of percentages error (% error) and percentages RSD (% RSD), respectively. The values for accuracy and precision are acceptable according to ICH guidelines [11, 12] as seen in Table 2.

**Table 2:** Intra-day and inter-day precision and accuracy of MST (QC) samples

Day of analysis	MST measured concentration		
	LQC (15 ng/mL)	MQC (50 ng/mL)	HQC (150 ng/mL)
Day 1	15.06	48.40	152.68
	14.81	47.92	152.32
	14.77	47.98	151.63
	14.79	48.08	152.56
	14.62	48.36	151.58
	14.73	48.20	148.58
	14.59	47.80	152.02
	14.61	47.94	150.70
	14.68	47.83	152.89
	14.70	48.04	152.10
	14.65	47.46	151.61
	14.50	47.87	151.63
Day 2	14.56	47.90	151.42
	14.59	47.36	150.52
	14.74	46.78	150.20
	14.63	46.68	148.79
	14.67	46.77	149.41
	14.72	46.64	149.89
Day 3	14.50	46.84	150.12
	14.42	46.51	148.57
	14.47	46.62	149.65
	14.42	46.34	147.33
	14.50	46.66	147.88
	14.50	46.34	147.79

	LQC (15 ng/mL)		MQC (50 ng/mL)		HQC (150 ng/mL)	
	Intra-day assay^a^	Inter-day assay^b^	Intra-day assay	Inter-day assay	Intra-day assay	Inter-day assay
Mean	14.71	14.63	47.99	47.39	151.69	150.49
Standard deviation (SD)	0.14	0.15	0.26	0.71	1.15	1.69
Precision (%RSD)	0.95	1.01	0.54	1.49	0.76	1.12
Accuracy (%RE)	− 1.93	− 2.43	− 4.02	− 5.22	1.13	0.33

^a^Average of twelve determinations of day 1

^b^Average of six determinations in three consecutive days

#### Stability

Stability studies of MST under different conditions were perfumed using quality control samples. The deviation of the results from the mean value of the samples was less than 1.53% for RLMs. No observed loss of MST was found during sample storage and handling under the examined conditions. Stability results (Table 3) indicated that RLMs matrix samples containing MST can be kept under laboratory conditions with no observed loss of MST.

**Table 3:** MST Stability data in *RLMs* matrix under different conditions

Nominal concentration (ng/mL)	Mean (ng/mL)	Standard deviation (SD)	Precision (RSD%)	Accuracy (RE%)
Room temp. for 8 h				
15	14.80	0.15	0.99	− 1.34
50	48.16	0.20	0.41	− 3.69
150	151.56	1.53	1.01	1.04
Three freeze–thaw cycles				
15	14.62	0.07	0.50	− 2.52
50	47.82	0.20	0.42	− 4.36
150	151.82	0.72	0.47	1.22
Stored at 4 °C for 24 h				
15	14.65	0.07	0.49	− 2.32
50	47.02	0.50	1.07	− 5.96
150	150.04	0.91	0.61	0.03
Stored at − 20 °C for 30 days				
15	14.47	0.04	0.27	− 3.55
50	46.55	0.19	0.42	− 6.89
150	148.56	1.11	0.75	− 0.96

### Metabolic stability study

**Fig. 4:**
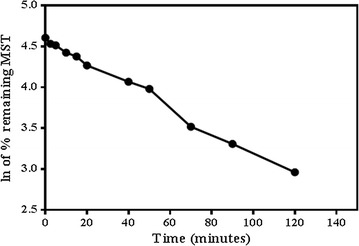
The metabolic stability profile of MST after incubation with RLMs. Metabolic reaction was stopped at different time points

After quenching the metabolic reaction using ACN at specific time intervals, concentrations of MST in RLMs matrix were computed. Plotting the ln of the % remaining MST (with respect to zero time) against incubation time was done as shown in Fig. 4. The first linear part of the curve showed a regression equation of Y = − 0.01378*X + 4.579 that was used for computing of in vitro t_1/2_ [16] (where in vitro t_1/2_ = ln2/slope). The slope was 0.01378 and therefore in vitro t_1/2_ was found to be 50.38 min. Consequently, CL_int_ was computed following in vitro t_1/2_ method [17] and found to be 3.11 ± 0.2 as shown in the following equation.

$$ CL_{int, app} = \,\frac{0.693}{{in vitro t _{1/2} }} .  \frac{\text{mL incubation}}{\text{mg microsomes}} .\frac{{45\,   {\text{mg microsome}}}}{\text{g liver}} .\frac{{20\,   {\text{g liver}}}}{\text{kg per body weight}} $$

$$ CL_{int, app} = \frac{0.693}{50.4} .  \frac{1}{1} .\frac{45}{12.5} .\frac{20  }{0.33} $$

$$ CL_{int, app} = 3 \,{\text{ml}}/{ \hbox{min} }/{\text{kg}} . $$

### Excretion of masitinib in rat urine

**Fig. 5:**
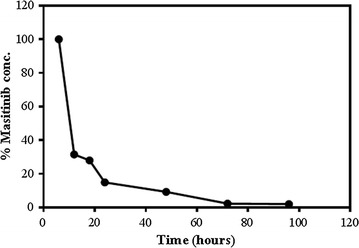
Excretion rate of MST in Sprague Dawely rats after single oral dose of 30.8 mg/Kg

Masitinib was found in rat urine after 6 h of dosing and began to decrease by time until almost disappeared after 96 h (Fig. 5). These concentrations equal to the % MST with respect to 6 h time (which represents 100%). The % MST in urine was plotted against collection time as shown in Fig. 5.

## Conclusion

Validated LC–MS/MS method was developed for assaying MST in RLMs matrices. The suggested method showed a linear dynamic range of 5–200 ng/mL with *LOD* and *LOQ* of 0.25 and 0.76 ng/mL, respectively in RLMS. Analysis time was lower than 5 min showing the rapidity of the method. The current method was applied with a great success for quantitation of MST in RLMs and the MST metabolic stability was further evaluated. The metabolic stability was expressed in terms of in vitro t_1/2_ (50.38 min) and CL_int_ (3.11 ± 0.2). The developed method was applied also to measure the rate of masitinib excretion in rat urine.
